# Modeling and Analysis of a Microresonating Biosensor for Detection of *Salmonella* Bacteria in Human Blood

**DOI:** 10.3390/s140712885

**Published:** 2014-07-18

**Authors:** Mahdi Bahadoran, Ahmad Fakhrurrazi Ahmad Noorden, Kashif Chaudhary, Faeze Sadat Mohajer, Muhammad Safwan Aziz, Shahrin Hashim, Jalil Ali, Preecha Yupapin

**Affiliations:** 1 Institute of Advance Photonics Science, Nanotechnology Research Alliance, Universiti Teknologi Malaysia (UTM), 81310 Johor Bahru, Malaysia; E-Mails: amadleven@gmail.com (A.F.A.N.); kashif.ali02@gmail.com (K.C.); safwan797@gmail.com (M.S.A.); jalilali@utm.my (J.A.); 2 Bioinformatics Research Group, Universiti Teknologi Malaysia (UTM), 81310 Johor Bahru, Malaysia; E-Mail: faezbio@yahoo.com; 3 K Economy Research Alliance (RAKE), Universiti Teknologi Malaysia (UTM), 81310 Johor Bahru, Malaysia; E-Mail: p-sharin@utm.my; 4 Advance Research Center for Photonics, Faculty of Science King Mongkut's Institute of Technology Ladkrabang, Bangkok 10520, Thailand; E-Mail: kypreech@kmitl.ac.th

**Keywords:** nonlinear optics, microring resonator, biophotonics, biosensor, *Salmonella* bacteria

## Abstract

A new photonics biosensor configuration comprising a Double-side Ring Add-drop Filter microring resonator (DR-ADF) made from SiO_2_-TiO_2_ material is proposed for the detection of *Salmonella* bacteria (SB) in blood. The scattering matrix method using inductive calculation is used to determine the output signal's intensities in the blood with and without presence of *Salmonella*. The change in refractive index due to the reaction of *Salmonella* bacteria with its applied antibody on the flagellin layer loaded on the sensing and detecting microresonator causes the increase in through and dropper port's intensities of the output signal which leads to the detection of SB in blood. A shift in the output signal wavelength is observed with resolution of 0.01 nm. The change in intensity and shift in wavelength is analyzed with respect to the change in the refractive index which contributes toward achieving an ultra-high sensitivity of 95,500 nm/RIU which is almost two orders higher than that of reported from single ring sensors and the limit of detection is in the order of 1 × 10^−8^ RIU. In applications, such a system can be employed for a high sensitive and fast detection of bacteria.

## 1. Introduction

*Salmonella Typhimurium* bacteria which cause thypoid fever contain flagellin (a protein with 494 amino acids of spherical shape, found in transparent filament cylinders). Several investigations have been conducted for the diagnosis of *Salmonella* such as electrochemical biomolecule detection, the TUBEX test, and the Felix-Widal test. These techniques requires labeling steps for isolation of the target molecules which takes additional time, and adds reagent costs and complexity [1].

The high demand for precise detection for biomolecules is increasing the tendency towards the improvement of biosensing techniques. Several techniques based on the change in phase or frequency such as Surface Enhanced Raman Spectroscopy (SERS), fluorescence, refractometry and microresonator biosensors have been introduced for the improvement of such detection in the past few years [2]. Most biosensors are focused on the label-free biosensing detection of bacterial proteins such as quartz crystal microbalance (QCM), surface plasmon resonance (SPR), coupled plasmon-waveguide resonance (CPWR) spectroscopy, and optical waveguide light-mode spectroscopy (OWLS) [3]. Label-free detection provides direct detection of biomolecules in natural forms without any adding any impurity to the target which results in performs detections with high sensitivity and low equipment costs.

Recently, the optoelectronics biosensor based on a microresonator in a photonics circuit has been introduced in the biosensing field as an important device that provides the high efficiency in biomolecule detection [1]. The microring resonator (MRR) is the main component in a photonics biosensor circuit which confines photons within the detection system [4]. The MRR system can be operated with more accuracy and low power consumption and can be fabricated in small size at low cost [5]. The MRR is used as the optofluidic devices for biosensing [6], and chemical analysis [7] as it can be integrated within photonics circuits. Nowadays, numerous works have been conducted to achieve the optimum design, size and material for enhancing the sensing purposes in terms of modeling and experimental methods. A limit of detection (LOD) on the order of 10^−6^ RIU is reported for integrated interferometer sensors with large interaction lengths [8,9]. Resonant-based sensors have also proved as ultra-sensitive sensors due to their high quality factor and sharp resonant peaks which provide a narrow detectable spectral shift. Using a slot waveguide-based ring resonator contributes to increase the light-matter interaction which improves the sensor's sensitivity to 298 nm/RIU and the LOD to 10^−5^ RIU [10]. Thanks to the Vernier effect, biosensors consisting of cascade ring resonators can also provide sensitivities as high as 2169 nm/RIU and LOD on the order of 10^−6^ [11]. Based on the size of the microresonators, design and waveguide material, the sensitivity of resonant-based sensors can be enhanced to an ultra-sensitive value of 10^5^ nm/RIU [12].

In this work, a novel photonics biosensor configuration comprising a Double-side Ring Add-drop Filter microring resonator (DR-ADF) made from SiO_2_-TiO_2_ material is proposed for the detection of *Salmonella* bacteria (SB) in blood. *Salmonella* total antibodies are applied on a thin film flagellin layer and loaded on the sensing and detecting microring resonators and used as the probe for the detection of SB. The nonlinear treatment of the soliton pulse due to the Kerr effect inside DR-ADF is studied analytically. We achieve an ultra-high sensitivity detection of 95,500 nm/RIU and a very low limit of detection in the order of 1 × 10^−8^ RIU, which are almost two orders better than those reported for single ring sensors. In applications, such a system can be employed for a high sensitive and fast detection of bacteria. The main advantage of our proposed sensor is that it satisfies both intensity and wavelength interrogation approaches in comparison with other optical sensors and can be employed for a high sensitive and fast detection of *Salmonella* bacteria.

## 2. Sensor Layout

**Figure 1.**
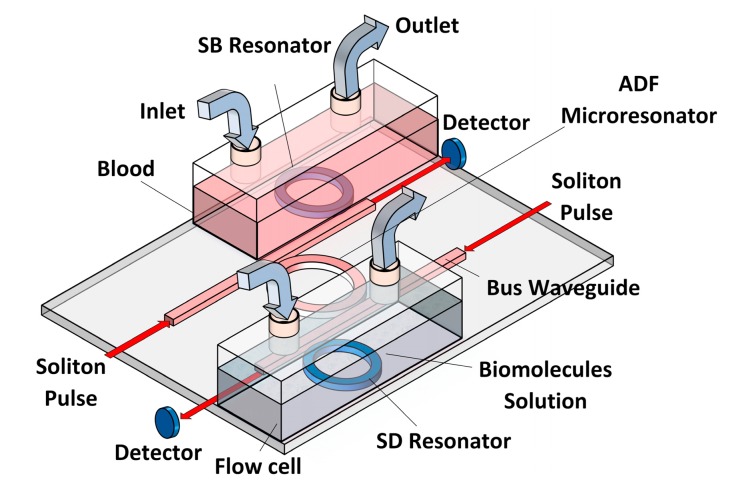
Add-drop filter microresonator with two side rings as biosensing probe inside OWLS flow cell.

Based on the spectroscopic method known as optical light-mode spectroscopy (OWLS), the bio-molecule must be inserted into a flow cell at the inlet and flow out through the outlet. The small change in the refractive index changes the output intensity signal due to the absorption of pulse radiation by the biomolecules [13]. Figure 1 shows the suggested design for OWLS flow cells. This design consists of a double flow cell on which embedded single rings at the through and drop ports of the microresonator system have been installed.

The add-drop filter (ADF) [14] microring resonator system is attached into two single ring resonators called baseline resonator and detecting resonator. The middle add-drop resonator is used to form the interference between the entrance signals from baseline and detection rings which increases the sensitivity of proposed sensor rather than ADF sensors. *Salmonella* total antibodies are applied on the thin film flagellin layer and loaded on the baseline and detecting microring resonators and used as the probe for the detection of SB. In practice, the adsorption of *Salmonella* flagellin on the surface of the sensing resonator causes a change of effective refractive index in which brings about the shift of signals circulating in the interferometer ring.

The flagellin sample layer for *Salmonella* bacteria can be prepared by heat-induced depolymerization of the flagellar filament by incubation at 65 °C for 15 min. The waveguide (microring system) is placed into the head assembly of an OWLS optical scanner attached with the flow-through cuvette (flow cell) [15]{Delezoide, 2012 #2136}. In order to provide the specific and selective binding of the *Salmonella* bacterium into the flagellin layer, *Salmonella* total antibody is applied on the flagellin sample layer which is loaded on the ring waveguide. *Salmonalla* total antibody including *Salmonella paratyphi* A H Ab, *Salmonella paratyphi* B H Ab, *Salmonella typhi* H D Ab, *Salmonella typhi* O Vi Ab, and *Salmonella typhi* O D Ab is used for selective detection of Salmonella H, Type A, B, C and Salmonella O, Type Vi, and Salmonella O, Type D, respectively [16]. A specific and selective binding of the *Salmonella* bacterium can be obtained as SB can only reacts with its own *Salmonalla* total antibody. Thus, the detected wavelength shift due to the variation of the refractive index is specifically due to the reaction between antigen (*Salmonella*) and antibody.

**Figure 2.**
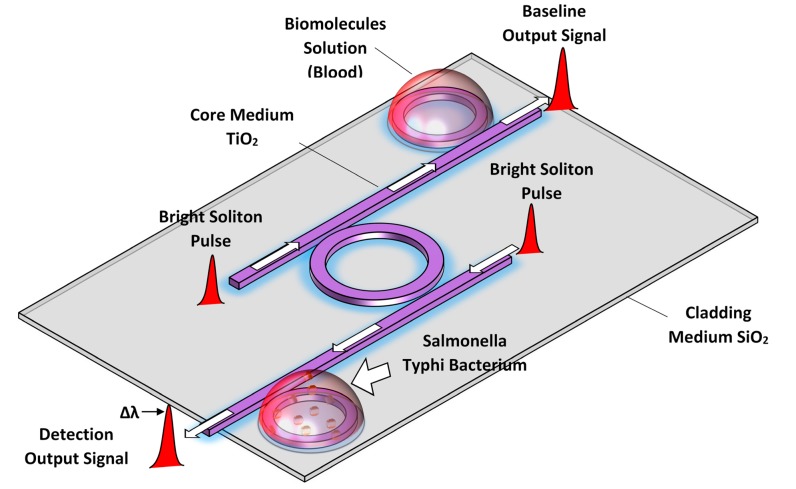
Diagram of biosensor consist of DR-ADF microring resonator within biomolecules solution.

The baseline resonator is placed within the blood with no SB for the reference signal (*i.e.*, base line signal) of the output intensity and the detecting resonator is embedded inside the flow cell which contained blood with *Salmonella* bacteria. The detector is placed after the baseline and detecting units. In Figure 2, the sensing moment is illustrated by both single rings for detection proposes. In this configuration, the baseline and detecting units are placed within in two separated flow cells and able to reduce the detection time since the sensing operation for the baseline and detecting units can be conducted simultaneously.

The waveguide parameters used in this modeling based on the TiO_2_ as the core medium with refractive index 2.00 and the SiO_2_ as the cladding with refractive index 1.75 for the MRR system [17]. The average refractive index of *Salmonella* flagellin layer with thickness of 2.2 nm on the surface of the waveguide is *n*_3_ = 1.43 [3]. Two types of solutions are used for aqueous medium in modeling parameters. The concentration of 30 (g/L) of oxygenated and oxygenated hemoglobin solution with the refractive index of 1.339 [18] is used as the baseline solution (blood) and the concentration of 1 mg/mL *Salmonella* solution with refractive index of 1.33133 [3] used as contaminated blood in the detecting unit. The refractive index is changed due to the reaction between SB with its total antibody applied on the surface of the microresonator.

The optical soliton pulse laser is launched to the MRR system through the input port. The pulse travels inside the bus waveguide and a portion of the pulse couples into the ring resonator. For the baseline and detecting units, the outer refractive index (cladding material) is changed due to specific reaction between antigen (*Salmonella*) and its antibodies. The biomolecule solution undergoes a molecular binding process with the cladding material at the ring surface which changes the refractive index while the pulse propagates within the ring [19]. At the output ports, a change in intensity and a shift in wavelength are observed due to the change in the refractive index which determines the sensor sensitivity.

The scattering matrix method is used for the analytical formulation to analyze the behavior of the electric field propagation inside the MRR system. The baseline unit flow cell is contained blood sample with no SB whereas the detecting units flow cell contains the contaminant blood (mixed with SB). The change refractive index of these two biomolecule solutions (*i.e.*, blood samples with and without SB) is the key parameter for the sensing. The *Salmonella* flagellin refractive index is considered as 1.43 for a thickness of 2.2 nm flagellin from the waveguide surface based on the previous research [3]. The radius of ADF micro resonator is 320 μm and the radius of the baseline and detecting microring resonators is150 μm. The real part of blood refractive index is expressed as:

(1) $$n=n_{H_{2}O}+\alpha C$$

where *n_H_*_2_*_O_* is the refractive index of distillate water, and *C* shows the hemoglobin concentration and α represents the specific refraction increment. The refractive index of whole blood can be estimated using the Gladstone–Dale equation *n_blood_* = *n_e_V_e_* + *n_p_V_p_* where *n_e_* and *n_p_* are the refractive indices of erythrocytes and plasma, respectively, and *V_e_* and *V_p_* are the corresponding volume fractions [18]. The simulation provides the different output pulse intensities through the microresonator between the baseline and detecting resonator, which shows the presence of the SB inside the blood due to change in the refractive index of the system. The Figure 2 shows the operational diagram of the DR-ADF microring resonator system.

The wavelength of 632.8 nm has been used for studying blood in the visible range. A soliton laser is used as the input pulse of the sensing due to the signal stability of solitons [20]. Another advantage is the confinement of the optical intensity within the waveguide due to the nonlinearity effect as compared to other lasers. The generation of the red soliton laser has been reported by Kudlinski *et al.* which has been employed for the blood analysis [21].

**Figure 3.**
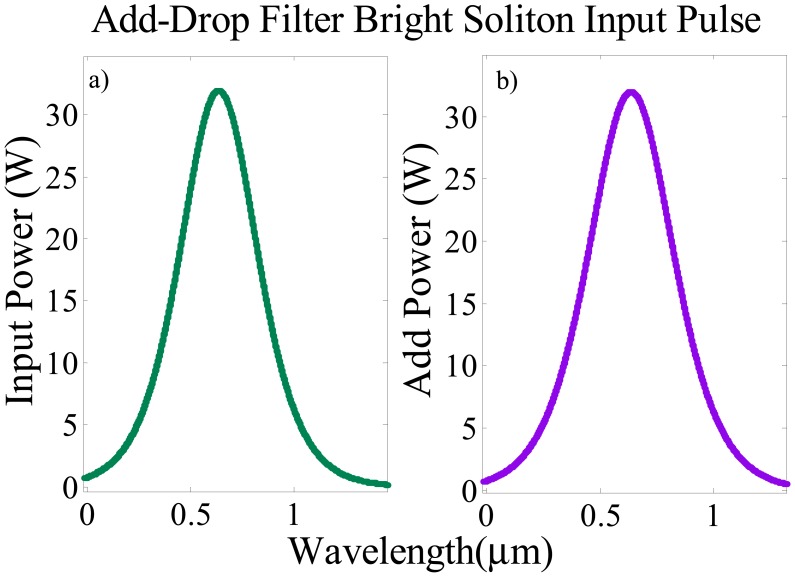
(**a**) Input port bright soliton pulse; (**b**) Add port bright soliton pulse.

The bright soliton pulses as the input source are launched at the input and add ports of the system. The equation of the optically bright soliton pulse is expressed [22] as:

(2) $$E_{in}=E_{\text{Bright}}=A\sec h\left(\frac{T}{T_{0}}\right)\text{exp}\left[\left(\frac{x}{2L_{D}}\right)-i\omega t\right]$$

which where *A* is the optical power amplitude, *x* shows the propagation distance, *T* represents the soliton pulse propagation time, and *T* = *t* − *β*_1_*x* is a soliton pulse propagation time in a frame moving at the group, and *T*_0_ is the initial propagation time which is equal to soliton pulse width. *L_D_= T*_0_/*|β*_2_*|* shows the dispersion length of the soliton pulse, ω is the frequency shift and *t* is the phase shift, where *β*_1_ and *β*_2_ are the coefficients of the linear and second-order terms of Taylor expansion of propagation constant [22]. The propagation of the soliton pulse within the system shows the balanced between the dispersion and the nonlinearity effect which are group velocity dispersion and the self-phase modulation respectively. The bright soliton pulses are simulated in Figure 3 *versus* wavelength with the center wavelength 632.8 nm. Two identical bright soliton pulses are launched into the input and add ports as shown in Figure 3a,b.

The analytical formulation is conducted based on the transfer matrix method to determine the transfer function of the electric field propagation inside the system. The inserted pulses at both ports propagate within the bus waveguide and a portion of the pulse (evanescent field) is coupled into the center ring resonator as *E*_1_ with cross coupling $iS_{1}=\sqrt{(1-\gamma_{1})k_{1}}$

Whereas, the other portion of the pulses from the input and add port travel through the bus waveguide to the through port and drop port respectively with self-coupling coefficient $C_{1}=\sqrt{\left(1-\gamma_{1})(1-k_{1}\right)}$

The center ring of the ADF microring resonator acts as a signal filter. The self and cross coupling depends on the parameters coupling coefficient *k*_1_ and the coupler loss *γ*_1_ [23]. The pulse *E*_1_ propagates in clockwise direction along the ring and interferes with the portion of bright soliton pulse, *E*_3_, inside the ring resonator. Using the scattering matrix method, the relationship between the output and input port's electric fields of the ADF microring resonator can be determined as:

(3) $$\left(\begin{array}{c} E_{1}\\ E_{\text{out}} \end{array}\right)=\left(\begin{array}{cc} C_{1} & iS_{1}\\ iS_{1} & C_{1} \end{array}\right)\left(\begin{array}{c} E_{4}\\ E_{in} \end{array}\right)$$

(4) $$\left(\begin{array}{c} E_{3}\\ E_{\text{drp}} \end{array}\right)=\left(\begin{array}{cc} C_{2} & iS_{2}\\ iS_{1} & C_{1} \end{array}\right)\left(\begin{array}{c} E_{2}\\ E_{a} \end{array}\right)$$

**Figure 4.**
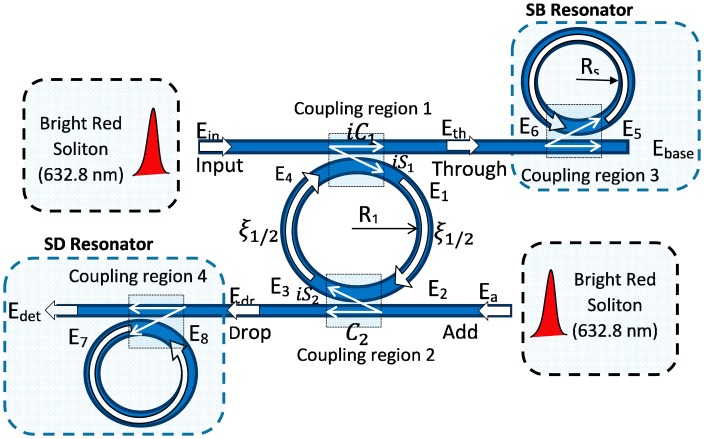
Schematic diagram of the DR-ADF microring resonator.

The output signal at the through and drop port are inserted into the baseline and detecting units, separately as shown in Figure 4. The baseline resonator provides the reference signal whereas the detecting resonator operates as the detection unit for the presence of SB in the bio-solution.

As the pulse travels in the bending waveguide (ring waveguide), the round trip loss *α* and phase shift *ξ*_1/2_ = exp(−*αL*/4−*iKnL*/2) changes [24], and the relationship between the propagating fields inside the center rings̶ can be determined as:

(5) $$\left(\begin{array}{c} E_{2}\\ E_{4} \end{array}\right)=\left(\begin{array}{cc} \xi_{1/2} & 0\\ 0 & \xi_{1/2} \end{array}\right)\left(\begin{array}{c} E_{1}\\ E_{3} \end{array}\right)$$

where *α* is the linear attenuation coefficient, *L* is the circumference of the ring resonator and *K* represents the vacuum wavenumber [23,25]. The refractive index *n* of the light pulse which propagates within nonlinear medium can be written as:

(6) $$n=n_{L}+n_{NL}\cdot I=n_{L}+n_{NL}\cdot \frac{P}{A_{\text{eff}}}$$

where *n_L_* is linear refractive and *n_NL_* is nonlinear refractive index. The intensity is shown by *I*, the optical power is shown by *P* and, *A_eff_* is the effective mode core area of waveguide and *P*. From Equation (6), the nonlinear Kerr effect is inside the system due the change of refractive index *n* which causes the phase shift of the propagating pulse. The biomolecules inside the biomolecule solution establish a molecular binding matrix with the ring cladding that results in a change in the effective refractive index, *n_eff_*, which can be determined as:

(7) $${n_{\text{eff}}}^{2}=\beta {n_{cr}}^{2}+\left(1-\beta \right){n_{cd}}^{2}$$

where *n_cr_* is the core refractive index, *n_cd_* is the cladding refractive index and *β* shows the propagation constant where *α* is linear attenuation constant and *k* = *2π*/*λ* is the vacuum wavenumber [26]. From Equation (3) to Equation (5), the relationship between the output electric field *E_out_* with respect to the input fields can be written as:

(8) $$E_{\text{out}}=E_{in}\left[\frac{C_{2}-C_{1}\xi }{1-C_{2}C_{1}\xi }\right]-E_{a}\left[\frac{S_{1}S_{2}\xi_{1/2}}{1-C_{2}C_{1}\xi }\right]$$

(9) $$E_{dr}=E_{a}\left[\frac{C_{2}-C_{1}\xi }{1-C_{2}C_{1}\xi }\right]-E_{in}\left[\frac{S_{1}S_{2}\xi_{1/2}}{1-C_{2}C_{1}\xi }\right]$$

The electric field at the baseline and detecting units can be obtained:

(10) $$E_{\text{base}}=E_{th}\left[\frac{C_{3}-\xi (1-\gamma )}{1-C_{3}\xi }\right]$$

(11) $$E_{\det}=E_{dr}\left[\frac{C_{4}-\xi (1-\gamma )}{1-C_{4}\xi }\right]$$

The output intensity at through *I_out_* and drop *I_drp_* ports can be obtained as:

(12) $$I_{\text{base}}=\left(E_{\text{base}}\right)\left({E_{\text{base}}}^{*}\right)={\left|E_{\text{base}}\right|}^{2}$$

(13) $$I_{\mathrm{detc}}=\left(E_{\mathrm{detc}}\right)\left({E_{\mathrm{detc}}}^{*}\right)={\left|E_{\mathrm{detc}}\right|}^{2}$$

## 3. Results and Discussion

The input pulse consists of a bright soliton with wavelength 632.8 nm that is fed into the ADF microring resonator system as shown in Figure 4. The radius of the ADF ring *R*_1_ is 320 μm and the baseline and detecting microresonators have equal radii of *R_s_* = *R_d_* = 150 μm. In the simulation, the following optical parameters are used: coupling coefficient ratios *k*_1_:*k*_2_ = 50:50, *k*_u_:*k*_d_ = 50:50, *A_eff_* = *0*.10 μm^2^, *n*_2_ = 4.2 × 10^−17^ m^2^/W [27], *α* = 0.4 dB/cm [28] and the lossless coupling, *γ* = 0, is supposed for the proposed system [29].

**Figure 5.**
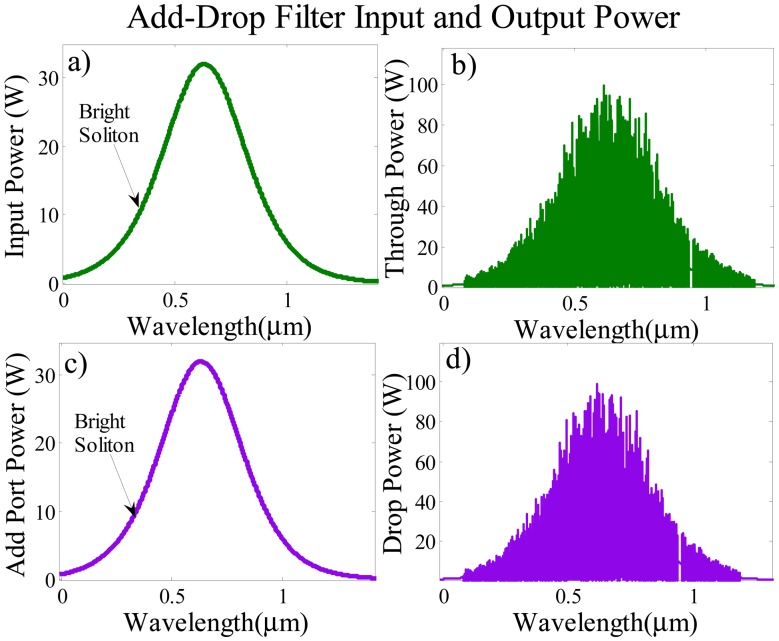
Schematics diagram of DR-ADF microring resonator.

The output power at the through and drop ports of the system using the iterative method for 20,000 roundtrips are shown in Figure 5b,d. The chirped output signals are obtained at the through and drop ports due to nonlinearity effect as the pulse travels for 20,000 roundtrips which is the recommended distance to enhance nonlinearity effects [30].

In Figure 5 the output power at both through and drop ports are amplified up to 100 W from the 32 W bright red soliton input pulse due to the constructive interference in the centre ring with the incoming input pulse coupled from the bus waveguide. A uniform set of optical parameters including identical coupling coefficients, and coupler loss are used for the main ADF resonating system to attain similar output signals from through and drop ports as shown in Figure 5b,c. The similarity in the output filtered signals show the self-calibration of the sensing system at the through and drop ports.These identical chopped signals are launched simultaneously into the detection and baseline units which act as sensing probe for the proposed biosensor.

**Figure 6.**
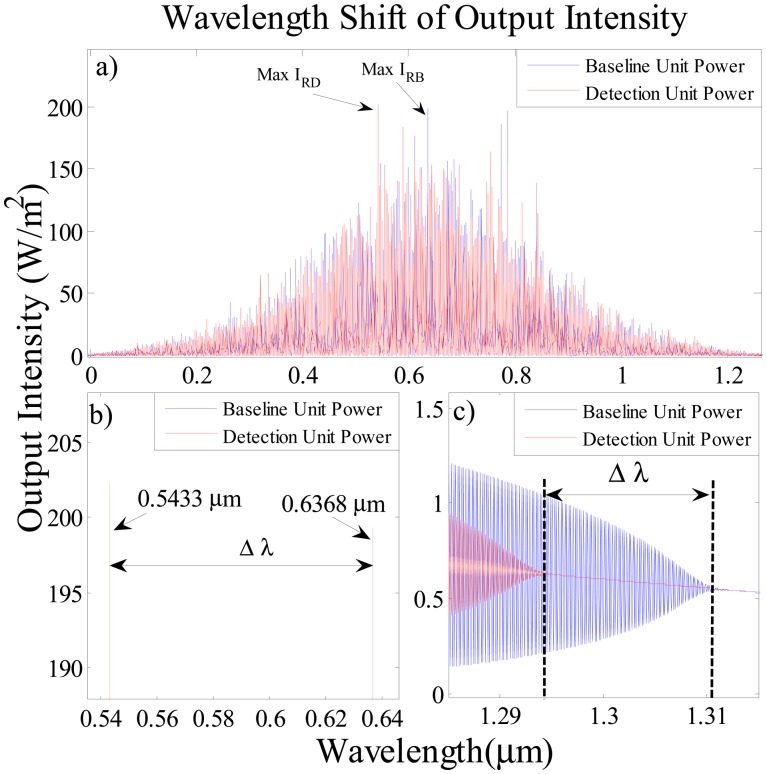
Wavelength shift on baseline cause by the *Salmonella* bacteria effect on output intensity.

Figure 6 shows the output signals at the baseline and detection ports. The bright red soliton pulse propagates within two side rings of ADF resonator which is immersed in the layer of human blood with and without *Salmonella*. In Figure 6, the output intensities at the detection and baseline ports are simulated for the wavelength range from 0 nm to 1200 nm where the blue lines represent the output intensity of the baseline ring resonator at the through port for blood samples without SB and the red lines show the output intensity of the detecting ring resonator at the drop port for contaminated blood. The Figure 6a shows the change of nonlinearity due to the presence of *Salmonella* bacteria and its effect on the overall output intensity of input solitons in the baseline and the detecting units. As demonstrated in Figure 6a the maximum resonance intensities for baseline and detection port are labeled as max I_RB_ and max I_RD_ respectively. The obtained intensity at the detection port with SB is 202.3 W/m^2^, which is higher than the intensity of the baseline unit at 199.3 W/m^2^.

Generally, microring resonators are studied using two interrogation approaches; the change in intensity and shift in wavelength [31]. In terms of intensity, an increase in the signal intensity in the presence of SB in blood is identified by 3 W/m in comparison to the blood without SB infection. Figure 6b,c is the enlargements of Figure 6a in the range 534.98 nm to 645.72 nm (visible region) and 1284.98 nm to 1315.03 nm (infrared region), respectively. Figure 6b shows the shift in the wavelength of maximum output peak towards shorter wavelength, Δλ = 95.50 nm, which is obtained from the difference between the max I_RB_ at 636.8 nm and the max I_RD_ at 543.3 nm.

The baseline and detecting units signals in Figure 6c indicate that the last point of bistablity [25] for blood without SB is at 1311 nm with the intensity of 0.551 W/m^2^ while for detecting unit it is at 1295 nm with intensity of 0.638 W/m^2^. Thus the wavelength shift and intensity variation in the infrared regime are 16.00 nm and 0.086 W/m, respectively. The DR-ADF microrng resonator provides a 95.50 nm shift in wavelength Δλ between baseline and detection output resonance intensity which is better than that if a conventional ADF biosensor [32]. The interaction length between light and biomolecules is proportional to sensitivity [1]. The DR-ADF microresonator provides a longer interaction length between light and biomolecules which brings about higher sensitivity. The measured interaction length of the proposed system is larger than that of other conventional waveguide biosensors [33,34] as it operates in 18.85 m along 20,000 roundtrips. Based on Equation (7) the change in refractive index is 9.9 × 10^−4^ which leads to achieving ultra-high-sensitivity detection of 95,500 nm/RIU. This sensitivity is higher than that of the microsphere resonating biosensor [35]. The limit of detection is in the order of 1 × 10^−8^ RIU which is better than that of the Vernier cascade ring biosensor [11].

**Figure 7.**
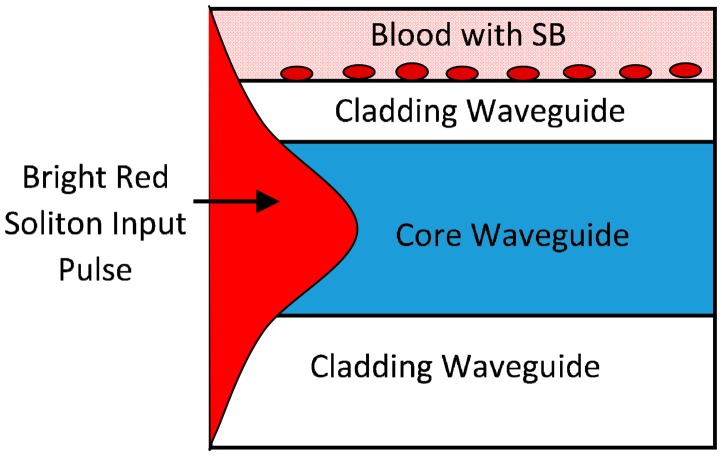
Schematics diagram of the bright red soliton pulse propagation.

The shift in the wavelength due to the presence of SB in the blood is caused by the interaction of the optical evanescent field with the external molecules on the top surface of the cladding waveguide as shown in Figure 7. The thin film flagellin layer coated on the cladding waveguide and *Salmonella* total antibodies are applied on this flagellin layer. Among the various kinds of biomolecules in the test sample (blood with SB) only *Salmonella* bacteria can bind with *Salmonella* antibodies. This leads to a change in the refractive index of the waveguide and enhances the nonlinearity effect of the system which causes the shift in wavelength of output signal [36]. In the other words, the proposed system can detect the presence of SB in blood with the wavelength shift in the maximum peaks and the wavelength shift in the last points of bistability. The main advantage of our proposed sensor is that it satisfies both intensity and wavelength interrogation approaches in comparison with other optical sensors.

Figure 7 shows a schematic diagram of the red soliton pulse propagation within a rectangular microring waveguide. As the pulse passes through the core, the evanescent field of the pulse interacts with the cladding medium. Since a portion of the red soliton pulse (evanescent field) reaches the outer medium (cladding and the blood), the presence of the SB within the blood causes a change of the pulse behavior while travelling through it as compared to the normal blood. The interaction of the *Salmonella* flagellin molecules with the surface of the cladding provides the binding process between molecules which can heat the cladding surface and provides change the effective refractive index of the system [36]. Thus, the nonlinearity effect (Equation (7)) of the soliton pulse propagation in the system is enhanced. The resonances intensity of the system changes and shift of the maximum resonance intensity wavelength is due to the SB infection in human blood.

**Figure 8.**
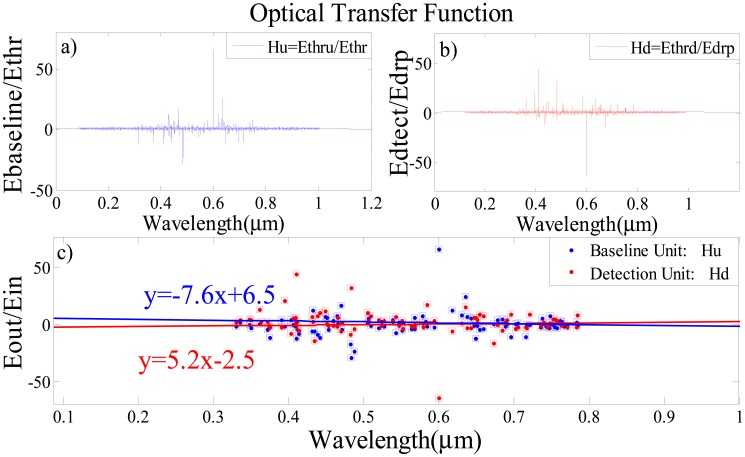
Optical transfer function for (**a**) base line unit (**b**) detection unit (**c**) both units with sensing line.

The optical transfer function (OTF), which is the output to input transmittance relation, *versus* wavelength for baseline and detection units are also simulated to obtain the sensing line for each unit. The OTF of baseline unit is demonstrated in Figure 8a which shows the relationship between the output signal from baseline unit (Figure 6a blue lines) and the launched filterd signals from ADF to this unit (Figure 5b). In the same procedure the relationship between the output signal from the detecting unit (Figure 6a red lines) and the launched filtered signals from the ADF into the detecting unit (Figure 5d) are simulated in Figure 8b. Two identical soliton signals are fed into the input and add ports of the sensor. Firstly, the signals are coupled into the middle ADF microresonator ring via *k*_1_ and *k*_2_ couplers. After meeting the resonant condition of the middle ADF ring, the output filtered signals are coupled into the baseline and detection single microresonators. The resonance conditions of the detection and baseline ring are changed based on the variation of the refractive index due to the attachment of *Salmonella* bacteria onto its antibody on the surface of the microresonators. This leads to a change in the spectrum of the optical transfer function for the baseline and detection resonators as shown in Figure 8a,b. In Figure 8c, linear least square curve fitting is used for OTF of the baseline and detecting units with considering the wavelength position of peaks points for the signals of each unit. The dots show the peak points that were measured from the changes in the refractive index due to the presence of SB, and the solid line represents the linear least square curve fitting for these points. The detection of SB can be tested by checking the slope variation of these sensing linear lines. As shown in Figure 8c for blood without SB, this line has negative slope (blue line) and the presence of the *Salmonella* bacteria brings about the change of slope from negative to positive.

The detection of the SB within blood is confirmed using the first order perturbation theory as the polarizability is proportional to the protein molecular weight [37]. The excess polarizability of *Salmonella* flagellin *α_ex_* = 4.48 × 10^−20^, *ε*_0_ is calculated by using *Salmonella* flagellin molar mass [33] which is used to characterize the *Salmonella* flagellin in the first order perturbation theory as *Δλ*/*λ* = *α_ex_σ_s_*/*ε*_0_ (*n*_1_^2^ − *n*_2_^2^) *R*. Here, *ε*_0_ represents the vacuum permittivity, *σ_s_* is the surface density for *Salmonella* flagellin [38], *n*_1_ is the refractive index of MRR medium, *R* is the radius of the ring and *n*_2_ is the refractive index of the *Salmonella* flagellin. The detection of the *Salmonella* bacteria from the expected wavelength shift with respect to the center wavelength of the input soliton is quite concordant with the expected molecular polarizability 1.5 × 10^−5^ which is obtained by *Salmonella* flagellin properties. The detected wavelength shift due to the variation of the waveguide refractive index is specifically due to the binding between antigen (*Salmonella* bacteria) and applied *Salmonella* total antibody on the loaded flagellin sample layer coated on the ring waveguide.

## 4. Conclusions/Outlook

In conclusion, a biosensing system consisting of microring resonators is introduced for detection of *Salmonella* bacteria in the human blood. The visible red soliton laser with center wavelength 632.8 nm is used as the input pulse for the biomolecule detection. The analytical formulation is conducted for 20,000 roundtrips pulse propagation in the microring resonator system by using the scattering matrix and iterative methods. The wavelength shift of the maximum resonance intensity is obtained in the presence of *Salmonella* bacteria. The simulation results show that a 95.50 nm shift towards shorter wavelength is obtained in visible range, and the shift in wavelength as 16.00 nm in the infrared range with resolution of 0.01 nm. The wavelength shift is due to the change in the effective refractive index caused by the SB in the blood. The main advantage of our proposed sensor is that it satisfies both intensity and wavelength interrogation approaches in comparison with other optical sensors and can be employed for a high sensitive and fast detection of *Salmonella* bacteria.

The authors would like to thank the Institute of Advanced Photonics Science, Nanotechnology Research Alliance, Universiti Teknologi Malaysia (UTM) and King Mongkut's Institute of Technology (KMITL), Thailand for providing research facilities. This research work has been supported by UTM's Tier 1(Q.J130000.2509.06H46)/Flagship (Q.J130000.2426.00G26 & Q.J130000.2431.00G29) research Grants.
